# Implementation of the Automated Medication Dispensing System–Early Lessons From Eswatini

**DOI:** 10.3389/ijph.2023.1606185

**Published:** 2023-10-12

**Authors:** Victor Williams, Samson Haumba, Fikile Ngwenya-Ngcamphalala, Arnold Mafukidze, Normusa Musarapasi, Hugben Byarugaba, Simbarashe Chiripashi, Makhosazana Dlamini, Thokozani Maseko, Nkhosikhona Advocate Dlamini, Clara Nyapokoto, Sharon Kibwana, Pido Bongomin, Sikhathele Mazibuko, Fortunate Bhembe, Sylvia Ojoo, Velephi Okello, Deus Bazira

**Affiliations:** ^1^ Center for Global Health Practice and Impact, Georgetown University, Mbabane, Eswatini; ^2^ Julius Global Health, Julius Center for Health Sciences and Primary Care, University Medical Center Utrecht, Utrecht University, Utrecht, Netherlands; ^3^ Center for Global Health Practice and Impact, Georgetown University, Washington, DC, United States; ^4^ Eswatini National AIDS Program, Mbabane, Eswatini; ^5^ Division of Global HIV and TB, Centers for Disease Control and Prevention, Mbabane, Eswatini; ^6^ Ministry of Health, Mbabane, Eswatini

**Keywords:** COVID-19, automated medication dispensing system, human immune-deficiency virus, integrated services delivery, client-centered care, non-communicable diseases, adherence

## Abstract

**Objectives:** This article describes the implementation of an automated medication dispensing system (AMDS) in Eswatini to increase medication access and presents the early lessons from this implementation.

**Methods:** The AMDS was installed at four health facilities across two regions through collaborative stakeholder engagement. Healthcare workers were trained, and clients who met the inclusion criteria accessed their medications from the system. Each step of the implementation was documented and summarised in this article.

**Results:** Early lessons suggest that implementation of the AMDS is acceptable and feasible to clients and healthcare workers and that phased introduction of medication classes, commencing with antiretroviral therapy (ART) and incorporating other medications in later phases is feasible. Additionally, improved client-centred messaging and communication, consistent power supply and internet network connectivity, and scheduling medication pickup with other services increase AMDS system utilisation.

**Conclusion:** Eswatini has many clients living with HIV and non-communicable diseases (NCDs). Easy, convenient, quick, non-stigmatising and client-centred access to ART and medication for NCDs is critical in addressing retention in care and achieving optimal treatment outcomes.

## Introduction

The Kingdom of Eswatini, a small country in Southern Africa of just over 1 million people, is burdened by a high prevalence of HIV infection, tuberculosis, and non-communicable diseases (NCDs). The 2020 Population-based HIV Impact Assessment (PHIA), also known as the Swaziland HIV Incidence Measurement Survey 3 (SHIMS 3), found that 24.5% of adults aged 15 years and above are estimated to be living with HIV, and the incidence of TB is estimated at 319 cases/100, 000 population [1, 2]. In 2020, Eswatini, together with Switzerland, were the first countries to achieve the UNAIDS “95-95-95” global “fast track” HIV targets in advance of the 2030 deadline [3] indicating that 95% of people living with HIV (PLHIV) in Eswatini knew their status, 95% who knew their status were on life-long antiretroviral therapy (ART), and 95% who were on ART had achieved HIV viral suppression. This achievement can be attributed to the commitment of the Government of the Kingdom of Eswatini and its effective collaboration with its multi-lateral and bilateral partners, including the United States President’s Emergency Plan for AIDS Relief (PEPFAR), the Global Fund to Fight HIV/AIDS, TB, and Malaria (GFATM), and UN agencies [4, 5]. Despite this success, challenges remain; epidemic control has not been achieved for some population subgroups such as adolsecent girls and boys, and young men and women; the prevalence of HIV among adolescents is increasing, with the burden of infection being disproportionately on female adolescents; and viral suppression rates are lowest in the age group 15–29 years [1, 4, 5]. HIV remains the country’s most common cause of death [4, 6]. Moreover, access to effective antiretroviral therapy has significantly decreased AIDS-related morbidity and mortality, allowing an increasing proportion of adults to survive into old age.

Eswatini, like other countries in sub-Saharan Africa, is undergoing an epidemiological transition from infectious diseases as the significant causes of morbidity and mortality to an increasing burden of chronic NCDs such as diabetes, cardiovascular diseases, mental health conditions, chronic kidney and pulmonary diseases and cancers [7–12]. Furthermore, as cohorts of PLHIV on ART age and the prevalence of NCDs among this population increase, the need for integrated care for HIV and NCDs is expected to increase [13]. This need is because health systems have been re-oriented to address the enormous burden of HIV effectively and are insufficiently prepared to handle the looming challenge of NCDs. We can leverage the chronic care systems developed for HIV for NCDs, and the integration of accessible and affordable clinical care and prevention of NCDs with HIV programs strengthens the capacity and capability of the health systems to address the full range of needs of PLHIV at both the community and facility level [14, 15].

More recently, the COVID-19 pandemic necessitated adaptations to service delivery systems to ensure PLHIV continued to receive care without undue interference from COVID-19 mitigation measures [16–19]. Some of the approaches successfully adopted in Eswatini include 3–6 months multi-month dispensing of antiretroviral and anti-TB medications and community outreaches for integrated services delivery, including medication refills, TB and HIV screening, basic clinical assessments and laboratory tests [5, 20, 21]. These methods ensured clients continued to access needed services despite the constraints imposed by the pandemic on health systems.

In the last decade, various technological innovations have enhanced integrated health services. These innovations can be applied at any level of healthcare to improve outcomes [22–25]. One such technology is the automated medication dispensing system (AMDS) [22, 24–28], which is presently being piloted at selected health facilities in Eswatini as part of COVID-19 adaptations to increase access to medications and convenience for clients but also as a long-term differentiated service delivery model.

In Eswatini, the AMDS, locally called Lula Meds™, is a tightly controlled system for dispensing medications and uses an electronic cabinet system to provide clients access to their prescribed medication parcels. It includes an automated cabinet with a touchscreen monitor connected to secure storage cabinets. Medications for selected clients are prescribed, prepacked in parcels, and placed in the cabinets [26, 29]. Clients receive a one-time password (OTP) via short message service (SMS), providing access rights to the system and a specific cabinet with the client’s medication parcel [30]. The cabinets are temperature controlled, placed securely, and use SMS technology to inform clients about their next collection due date and send reminders. The client has up to 7 days to pick up their parcel; once a cabinet is empty, the central system is updated to load the medication parcel for another client. There is no time restriction to access the AMDS; hence, it is a convenient prescription pickup solution for clients who work in shifts and who prefer to pick up medication before or after their shifts, clients who do not want to queue at the pharmacy because of time constraints, and those who prefer to access health facilities after-hours. Additionally, the AMDS provides computer-controlled storage, dispensing, inventory management of medications, and client reminders, thus potentially saving pharmacists’ and doctors/nurses’ time. The financial modelling studies on AMDS implementation have shown a high return on investment in the time nurses and pharmacy technicians spent on medication-related work activities before and after the implementation of AMDS [25, 26].

The AMDS provides clients with quick and convenient prescription retrieval and access to medications for chronic care clients; it can reduce congestion at health facilities by reducing the number of clients queuing at the clinic or pharmacy and promoting adherence and continuity of care. The system also limits interaction between healthcare workers and clients, thereby serving as an efficient means of social distancing, one of the key COVID-19 prevention measures. The AMDS can be placed in the pharmacy or off-site and remote locations. It is fully accessible and can be controlled remotely by the pharmacist, reduces waiting time for clients at the pharmacy counter, and retrieving medications takes about 30–60 s. A 2021 study showed a 15% increase in prescriptions filled at the Vanderbilt pharmacy after installing an AMDS (™Script Center) [31]. In addition, a 2018 study indicated that increased prescription adherence “can have a tremendous impact on quality and length of life, health outcomes, and overall healthcare costs.” [32].

While AMDS has been introduced as a differentiated model of delivering ART in other countries across the Southern African region, with utopian views of improved client care and administrative efficiencies [26, 33–35], there is limited data on implementation to guide scale-up in other locations and integrated dispensing of non-ART medications. Therefore, this paper, which is the first in the series, aims to describe the implementation of the AMDS in a low- and middle-income country (Eswatini) using an implementation science approach, the steps taken to set up AMDS, the initial challenges and how these were addressed, and critical lessons.

## Methods

We used methods that promote understanding and influence the implementation of outcomes as well as integrate evidence-based practices into routine practice to improve the quality and effectiveness of health services.

### Stakeholders, Intervention Set-Up and Data Sources

The Eswatini Ministry of Health (MOH) collaborated with different stakeholders to implement Lula Meds^TM^ at four health facilities in two regions of Eswatini. Central level MOH and a lead technical Implementing Partner (IP) led the conceptualisation, resource mobilisation, stakeholder mobilisation, buy-in, and implementation design and documentation of lessons. After addressing all the design and implementation concerns within the central level MOH for consistency, the national AIDS control program and national stakeholders, engagements with sub-national stakeholders included the Regional Health Management Teams (RHMT) in the two pilot regions of Manzini and Lubombo, the health facility managers and health providers in the intervention health facilities and the AMDS vendor who coordinated experiential learning visits outside Eswatini that are implementing similar systems. The lessons from the experiential learning visits were presented to central MOH leadership and incorporated into the final implementation model and standard operating procedures. The collaborative approach enabled the different stakeholders to understand and appreciate the potential benefits of the intervention; gain insights into the design, feasibility, and requirements for implementing the AMDS and its limitations; identify stakeholder expectations and concerns and collectively design solutions to address them; and clarify the responsibilities of each stakeholder in the implementation of Lula Meds ^TM^. A Community of Practice (CoP) was established for healthcare workers from implementing facilities to share experiences and learn from each other continuously. The CoP met once a quarter. Each process adopted for the implementation of this system–healthcare workers engagement/feedback meetings, planning, procurement procedures, training and rollout, were documented in meeting minutes/reports, project workplan, procurement documents, standard operating procedures, AMDS specification and installation plan, health facility monthly and quarterly reports; and the RHMT Reports. These different documents serve as information sources for this paper.

### System Specification and Sourcing

**FIGURE 1:**
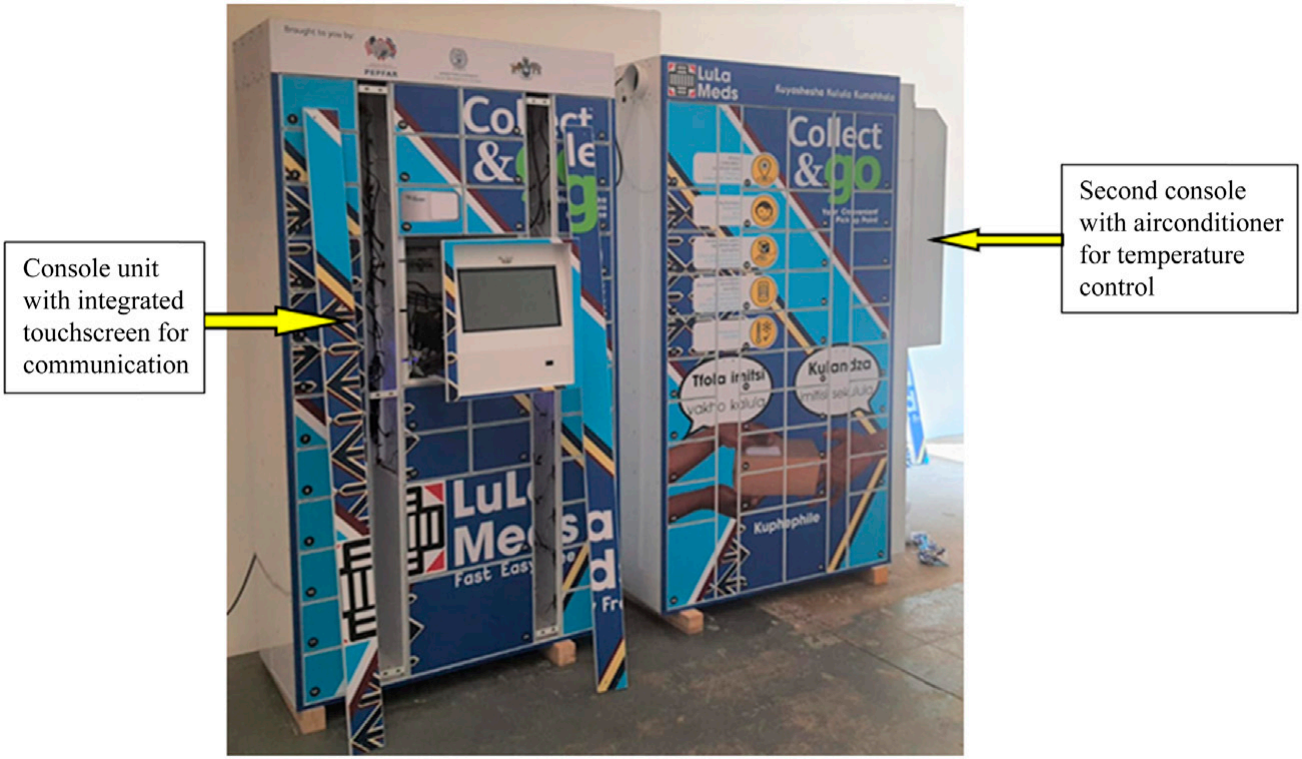
Automated medication dispensing system at installation showing the main console unit and the second unit with an air-conditioner (Implementation of the automated medication dispensing system in Eswatini. Eswatini, 2023).

The specifications of the AMDS are divided into hardware and software specifications. On the hardware front, the AMDS is composed of cabinets which are 1.15 m in length, 2.04 m in height and 0.515 m in depth. Two health facilities with more than 10,000 ART clients received three cabinets each, while the remaining two with less than 10,000 ART clients each received two. A cabinet comprises two units: a console unit that holds the pigeonholes used for the medication parcels, an integrated communications touchscreen used for opening the pigeonholes, and an air-conditioning unit that controls the temperature of the pigeonholes (Figure 1). The central console cabinet has 36 pigeonholes, while the second (standard) cabinet with air-conditioning has 40 pigeonholes. A standard cabinet composed of 40 pigeonholes can be added to the existing cabinet to expand the capacity of the cabinets, as was done in the case of the two health facilities with more than 10,000 clients.

The software component includes the cabinet operating software and a web application interface to register users for the AMDS. The web platform also has dashboards and reports for tracking the use of the cabinets and monitoring the system’s uptime. After a rigorous procurement evaluation criteria that included pricing, sustainability and successful installation and implementation in other countries, a vendor was identified (https://rightepharmacy.co.za/; https://collectandgo.co.za/about-collect-and-go/) was selected to provide the AMDS for Eswatini.

### Procurement and System Set-Up Planning

The procurement process was implemented in stages: the first stage was a scoping visit to the selected health facilities by the vendor. During the visit, the options for the system placement were discussed with facility management and healthcare providers. Space requirements in the proposed sites for the AMDS were defined. Health facility staff were oriented on the proposed technology, its benefits and limitations and solicitation of inputs was done. The second stage was the detailed costing of the system after the scoping visit, with costs divided into capital and operating costs. Capital costs included the main console cabinet, air conditioning cabinet, standard cabinet, and the cabinet and mobile software, including the configurations. Operating costs included yearly support fees, installation, training, branding of the AMDS, and transportation of the AMDS to each installation site. The vendor installed the AMDS at each health facility and trained healthcare workers to use the system. A vendor-supported pilot implementation was conducted over 3 months. Initial results from this pilot were used to refine the standard operating procedures for each health facility.

Four health facilities were selected for the initial implementation phase. The critical considerations for the selection of the facilities were location (rural-urban), type of facility (hospital, clinic, or dedicated ART centre), the willingness of the health facility management to accept the AMDS, the availability of accessible and safe space for the installation of the AMDS, and the volume of clients receiving HIV care. Therefore, two health facilities within urban centres each provide care for more than 10,000 clients on ART, while the other two were in semi-urban/semi-rural areas and provided care for less than 10,000 ART clients each. Although this system is designed for all chronic care clients, dispensing of ART was prioritised given that the HIV program has a well-controlled supply and accountability system of ART medications to pilot the system.

The installed system can hold a total of 384 medication parcels at any one time (equivalent to 384 pigeonholes), as follows: 116 pigeonholes that can hold a total of 116 medication parcels for health facilities serving more than 10,000 clients, and 76 pigeonholes that have a capacity for 76 medication parcels for health facilities serving less than 10,000 clients. Each health facility developed individualised strategies to enrol clients and the process flow.

### Personnel Training and Experiential Learning

Before the operationalisation of the AMDS, a pharmacist and nurse provider from each of the four sites, the national supply chain and ART coordinators were trained on how to operate and customise the AMDS. The training combined in-country and south-to-south experiential learning visits to health facilities and Call Centers in South Africa. During the experiential learning on the system, the visiting team was oriented to the set-up, client enrolment and process of resolving system malfunctions due to internet and electricity supply fluctuations. In addition, the South-to-South learning visit achieved other objectives, such as understanding how the AMDS functions and adaptations required for the Eswatini context, understanding possible challenges in the set-up and utilisation of the AMDS at the visited sites and how these were addressed, appreciating the roles and responsibilities of healthcare workers in the use of AMDS in designated facilities; understanding the standard operating procedures and how these can be adapted for each health facility; and learning about demand creation and recruitment strategies (marketing tools and health promotion) for AMDS for adaptation. On return, the team presented a report and key learnings to the MOH and stakeholders.

### Facility Multi-Disciplinary Team and Roles

A key step before rolling out the intervention was setting up the facility core team that would be responsible for the development of facility–specific standard operating procedures, client recruitment and redesign of client flow and overall implementation of the intervention. The roles of each team member are summarised in Table 1 below:

**TABLE 1:** Health facility core teams and their roles (Implementation of the automated medication dispensing system in Eswatini. Eswatini, 2023).

Core team member	Role
Clinician (medical officer/nurse)	Guides the recruitment of clients and ensures that clients are registered using the approved recruitment criteria. Guide the development of facility-specific standard operating procedures and client recruitment strategies
Pharmacist/pharmacy technician	Ensures that medicine parcels are loaded on time and medicine is dispensed according to prescriptions, coordinates monthly meetings, updates the team on the number of clients enrolled and suggests improvements to increase utilisation of Lula Meds ^TM^
Adherence Officer	Coordinates client flow, active recruitment and follow-up of enrolled clients who may have missed parcel pickup
Data Officer	Ensures updated reports on CMIS, correct documentation of client’s status and data summary of clients who have accessed services
IT/maintenance specialist	Ensures that the AMDS functions optimally (internet, software, and electricity). Troubleshooting and providing technical support to address operational issues with Lula Meds ^TM^

## Rollout of Services

### Eligibility Criteria for Clients

Implementation commenced in January–March 2022, and the AMDS was linked with ART service delivery. Eswatini provides ART differentiated services delivery (DSD models) for PLHIV in chronic care [5, 20, 21]. The eligibility criteria to access medication from the AMDS were aligned to DSD models for ease of implementation (Table 2).

**TABLE 2:** Eligibility criteria for picking medication from the automated medication dispensing system (Implementation of the automated medication dispensing system in Eswatini. Eswatini, 2023).

Criteria	Description
Clinical Status and Age	Clinically stable adults aged ≥18 years on select standard antiretroviral drug regimens
	Clinically stable NCD clients on ART
Unique identifier and contact details	Has a national ID number and/or client management information system (CMIS) number
	Client has a valid cell phone number
Adherence	Clients on ART for a minimum of 12 months at the health facility
	History of good adherence to HIV treatment (adherence ≥95%) and clinic appointments
	Clients with two consecutive suppressed viral load results, with the most recent within the preceding 6 month period
Comorbidity	Clients without current tuberculosis, opportunistic infections, or any adverse drug effects to current medications
	Clients without other chronic medical conditions requiring frequent clinical consultations
	Client is not on any injectable medication or liquid formulation

### Procedure for ART Client Enrollment on the AMDS

**FIGURE 2:**
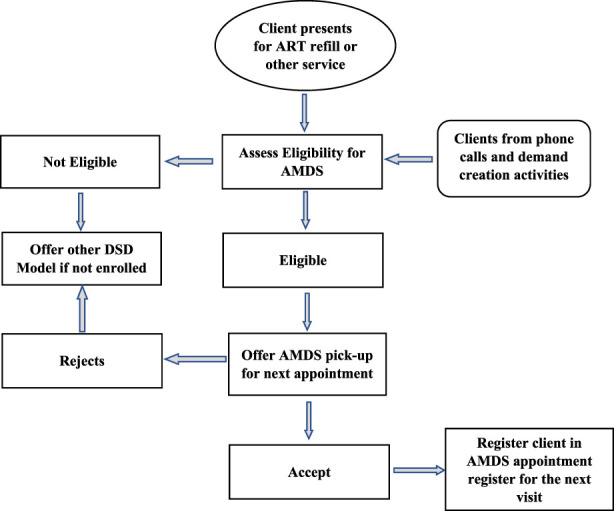
Enrollment process for clients on the automated medication dispensing system (Implementation of the automated medication dispensing system in Eswatini. Eswatini, 2023).

Eligible clients were systematically sought and informed about the AMDS by HCWs 3–6 months before enrolment. However, clients presenting at the select health facilities received information about the AMDS and health education through morning education sessions and information flyers before services commenced. The morning health education sessions are standard across health facilities where the providers update clients on changes in service delivery approaches, new treatment options and clarification on questions clients may have and hence were also used to inform most clients about the AMDS. Other opportunities used included clinical consultations, calls to clients when due for medication pickup, pharmacy dispensing points, and community drug distribution points. Different steps for client enrollment are described in Figure 2.

### Medication Type and Pick Up

**FIGURE 3:**
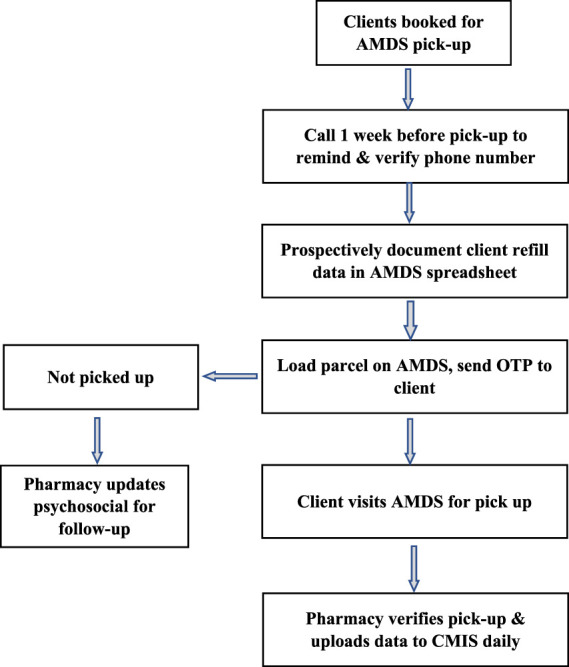
Process flow for medication pickup for scheduled clients (Implementation of the automated medication dispensing system in Eswatini. Eswatini, 2023).

The ART DSD models are classified into facility and community-based models. Stable clients on ART in Eswatini receive multi-month medication dispensing for 3–6 months, depending on the DSD model they are enrolled in. Available ART medications for stable clients depend on client needs; however, for the AMDS, facility teams preferred to enrol clients on a dolutegravir (DTG) based regimen. Dolutegravir is part of a triple-drug single-tablet antiretroviral regimen because of its high efficacy, resistance barrier, and few side effects. The medications are loaded in the AMDS for clients to access, and one of two approaches is used for AMDS dispensing. The first approach (Figure 3) enables prescheduled clients to visit a health facility for a medication pickup at any time within 7 days after the parcel has been loaded. Clients receive an SMS containing the one-time password (OTP) when their medication is ready for pick up, with two additional reminder SMS if they do not promptly pick up, the last one being 48 h before the OTP expires.

**FIGURE 4:**
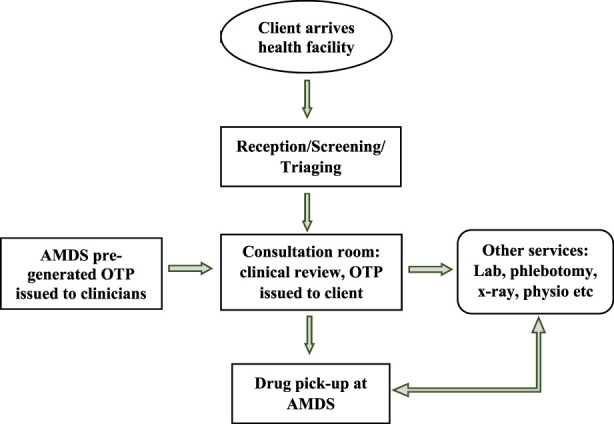
Approach two—process flow for medication pickup after clinical consultation (Implementation of the automated medication dispensing system in Eswatini. Eswatini, 2023).

In the second approach (Figure 4), mainly used to optimise the use of the AMDS, clinicians assign an OTP to clients who visit a health facility for a refill. This OTP is issued directly to the client to pick up their medication from the AMDS after clinical consultation with the clinician. This approach requires preloading the AMDS with a DTG-based regimen, the most common medicines the clinicians will prescribe. This approach relieves pressure on the dispensary.

For clients scheduled to pick up their medication from the AMDS, the pharmacy staff maintains a daily log of parcels with expired OTP. The clinicians initially review this to ensure they have not received their medication through some other model. Once this is confirmed, it is shared with adherence and psychosocial officers for follow-up. Clients who fail to pick up their medicines from the AMDS are assessed for barriers and returned to mainstream care to ensure adherence.

## Results

### Early Lessons From the Implementation of the AMDS

The AMDS has the potential to enhance services for clients with modifiable psychosocial barriers to on-time drug pickup, including lack of availability during clinic hours due to work, school, or other obligations. Early lessons are summarised under i) feasibility, acceptability, and utilisation; ii) demand creation and communication platforms for clients; iii) internet connectivity and interoperability; and iv) responsibility for implementation.

#### Feasibility, Acceptability, and Utilisation

a. Feasibility of implementation: Before the set-up of the AMDS, a thorough review of available resources, suitability of proposed implementation and supportive stakeholders is vital to successful implementation. The absence of any of these can hinder the set-up and operationalisation of the AMDS as it requires approvals and commitment from agencies, regulatory bodies, and implementation staff to ensure the different processes are implemented.
b. Acceptability of the intervention: a careful needs assessment before commencing intervention is necessary to identify if this proposed intervention meets the needs of the different stakeholders. This assessment will guide the implementers on context-specific modifications that will make the intervention acceptable to stakeholders.
c. Uptake of the intervention: the design of the intervention should be made with the end-users in mind. This will ensure the designed intervention meets their needs, increasing its uptake. Regular data reviews and formal/informal client engagement can identify end-users desires, which can guide several aspects of implementation, such as site selection, type of medication dispensed, operational hours and level of client support.

#### Demand Creation, Communication Platforms for Clients

a. Effective messaging will increase the utilisation of AMDS: a common observation across the four sites was that clients had challenges with the messages containing the OTP required to access medications from the AMDS. The message was long and only available in English, making it challenging for clients with a limited understanding of English who may not understand it. Some clients who mistakenly deleted the message or lost their OTP did not receive a second message or have another means of obtaining the OTP. This made clients resort to visiting health facilities directly to access their medication. Therefore, messages should be brief and available in the local language with an option to retrieve the OTP if the first message is lost.
b. Communication between facility staff and clients will increase utilisation: A mechanism or structure to ensure ongoing communication between clients and healthcare workers when they access their medication parcel is essential to identify clients’ needs and experiences. This will guide the facility core team to adjust services provided through theAMDS and ascertain if the client’s needs are still being met. A toll-free number for clients to call and request assistance can bridge this barrier.
c. Continuous sensitisation and offering the AMDS increase uptake: continuous sensitisation of clients on the inclusion criteria for AMDS and offering the service to eligible clients during consultation for other conditions can increase the uptake of the AMDS. In addition, synchronising visits for services such as drug pickup dates, viral load and cervical screening increases the uptake of AMDS by eligible women.

#### Network Connectivity and Interoperability With CMIS

a. Improved network connectivity will enhance the efficiency of the AMDS: internet connectivity and bandwidth posed a challenge for utilising the AMDS at all four sites. The internet may be poor or unavailable, causing delays in loading medication parcels for clients. An occasional power surge was Closely associated with this, leading to inconsistency in rebooting the system. A more reliable internet option and power backup system will ensure smooth operations.
b. Interconnectivity with the national reporting system will improve care: integration between the AMDS and the national MOH electronic reporting system (client management information system–CMIS) will ensure the synchronisation of appointments and tracking of clients’ access to care, further limiting duplication. This integration will limit the present manual system where CMIS is manually updated with AMDS data, as this does not allow real-time follow-up and intervention for clients who have missed a pickup.

#### Responsibility for Implementation

a. A focal staff to oversee operations will ensure optimal operations: although there is a focal team to coordinate the implementation of the AMDS at each health facility, a dedicated staff who oversees implementation is essential. This way, issues identified are followed up and resolved early. Limited human resource personnel to support the AMDS at one health facility with approximately 11,000 clients on ART hindered operations. This suggests a dedicated pharmacy staff may be required to support AMDS-related processes–parcel loading, OTP generation, reviewing pickup and generating line lists of clients whose OTPs expire for follow-up.
b. Non-client-based prescription cabinet loading is human resource effective: facilities that adopted approach 2 (Figure 4) had better acceptance of the AMDS by staff and resulted in more client enrollment.

## Discussion

This implementation science paper describes the process and early programmatic lessons from implementing the AMDS. A quantitative and mixed-methods study is underway and will provide information on the sociodemographic and clinical characteristics of clients accessing services at the AMDS, as well as perspectives of healthcare workers on the implementation and how it impacts their work. Additional lessons from these studies will inform scale-up to other health facilities and regions. Next steps for implementation include (a) translating the OTP message to the local language, simplifying it and making it possible for clients to access their OTP if they lose their phone or mistakenly delete the message; (b) exploring the procurement of a toll-free phone line through the MOH for clients utilising AMDS to enhance communication with healthcare workers and enable user feedback; (c) facilitate the engagement of the Health Management Information System (HMIS) and the AMDS vendors to assess the interoperability of AMDS with the CMIS to eliminate manual documentation, data upload and deduplication of clients accessing care; (d) integrating and operationalising access to NCD medications through the AMDS by identifying and enrolling stable NCD clients using any of the defined criteria and approaches that are suitable for the implementing health facilities and encourage implementing sites to explore the integration of other services in AMDS to mitigate the stigma associated with the use of AMDS. NCDs were not included in the first phase of implementation due to the erratic supply of NCD medications, but this has been addressed and NCD will be incorporated in the subsequent implementation phase; (e) explore the possibility of picking up medication from any AMDS cabinet regardless of the site to increase access to AMDS for mobile clients; (f) increase the utilisation of AMDS by reducing the duration a parcel stays in a cabinet (this will increase the number of clients that can be accommodated in this delivery model); (g) document client and healthcare worker experiences in the utilisation of AMDS and incorporate AMDS data review into routine performance monitoring at health facilities.

### Strength and Limitations

The implementation of AMDS follows a structured implementation science approach and has been approved by the Eswatini Health and Human Research Review Board (EHHRRB) and Georgetown University Institutional Review Board (GU - IRB). Implementation science seeks to systematically close the gap between what we know and what we do (often called the know-do gap) by identifying and addressing the barriers that slow or halt the uptake of proven health interventions and evidence-based practices [36]. The structured implementation will promote the systematic uptake of research findings and other evidence-based practices into routine practice scale-ups to improve the quality and effectiveness of AMDS and health services in general. Stakeholder engagements before, during and after implementation enable continuous improvement to meet the needs of different users.

As a limitation, lessons presented are mainly programmatic without data on patient outcomes. However, client-level health outcomes and the impact of AMDS on these outcomes will be documented and presented to strengthen the case for policymakers and implementers. A second limitation is the novelty of the intervention, which may take a while to reach most of those requiring it. In addition, clients without a mobile phone may be unable to access the intervention at the moment, and clients cannot pick up medication at a different site except where they registered. Notwithstanding these initial limitations, we are confident the ease of access to ART for some clients will increase positive feedback on this system.

### Recommendations

This paper describes programmatic lessons and success factors for implementing AMDS. Stakeholders and Implementers considering a similar intervention should review our implementation approach and early lessons to inform set-up. Given the potential of the AMDS to increase access to medications with a range of benefits to some clients, healthcare workers and health services delivery, the AMDS should adopted as a national strategy. In Eswatini, AMDS has been adopted as an official DSD model and formally launched by the Minister of Health. Other implementing partners and the MOH should mobilise resources for a national scale-up to make the AMDS accessible to residents of the other two regions of Eswatini and other PLHIVs who cannot access the intervention.

### Conclusion

Implementation of AMDS in Eswatini adopted an implementation science model that clearly defines the intervention, settings, the stakeholders involved, and the process for accomplishing the intervention. Since commencing the implementation, key lessons learnt have been incorporated into improving the AMDS, improving client-centricness, and increasing the demand for this technology. It is anticipated that further synthesis of the lessons and findings at the end of the study will augment the utility of AMDS as a new ART and NCDs DSD model for delivering ART and NCD medications and commodities.
